# Flourishing in Education: Psychometric Properties of the Flourishing Scale in a Sample of Romanian Teachers

**DOI:** 10.3390/bs14050366

**Published:** 2024-04-26

**Authors:** Beatrice Adriana Balgiu, Andrei Simionescu-Panait

**Affiliations:** Department of Career and Educational Training, National University of Science and Technology Politehnica Bucharest, 313 Splaiul Independenței, 060042 Bucharest, Romania; andrei.simionescu88@upb.ro

**Keywords:** validation, flourishing, positive affect, negative affect, mental health, teachers

## Abstract

The Flourishing Scale (FS) is one of the most well-known tools for assessing psychological flourishing. However, its psychometric properties have been little analyzed in the case of teachers. This study aimed to examine the validity of the scale in the case of a sample of Romanian teachers and to analyze the latter’s level of flourishing. In this regard, 323 Romanian teachers from the pre-university education system were recruited. Confirmatory factor analysis (CFA) was used to assess the construct validity of the scale, and Cronbach’s α and McDonald’s ω indices were used to assess internal consistency. The convergent validity was assessed by associating the FS with other instruments related to well-being: the Mental Health Continuum-Short Form and the Scale of Positive and Negative Experience. Network analysis was performed to examine the items that are particularly influential in the scale. As a result of the CFA, the one-factor structure of the scale was certified (χ^2^/df = 1.39; CFI = 0.99; RMSEA = 0.035). The internal consistency is excellent (both α and ω = 0.89). The FS correlates with both of the scales which operationalize components of well-being. The teachers’ flourishing level is above average. The network approach showed that the items related to self-acceptance, optimism, and respect had the highest indicators of centrality, and the item related to supportive social relationships was the least informative in the network. For the male subsample, flourishing means optimism about the future and respect for others, and for the female respondents, it is related to self-acceptance and respect. The results provide support for using the scale in assessing flourishing among teachers.

## 1. Introduction

The term flourishing—the highest level and optimal functioning in various domains—was approached both from a hedonic perspective (operationalized in terms of subjective well-being) and an eudaimonic perspective (the supreme happiness and sense of mastery). The eudaimonic perspective includes psychological models based on self-realization and personal growth [1,2,3,4], whereas the hedonic perspective includes models focused on life satisfaction, maximizing pleasure, and minimizing pain [4,5]. Studies suggest that the two perspectives are not totally different [6]. For example, Seligman [3] shows that the two concepts related to flourishing pertain to two different, but important, aspects of the general construct of well-being, while Keyes [7,8,9] attempted to identify the two perspectives, proposing an instrument called the Mental Health Continuum, which assessed the degree of positive mental health. 

One of the most popular instruments that operationalizes the eudaimonic well-being is the Flourishing Scale (FS) [4]. This measure has been translated into many languages, despite the fact that there are already a number of measurement instruments available to assess people’s degree of well-being. In order to evaluate the degree of flourishing of Romanian adults it is imperative that the structure of the scale be analyzed in the Romanian language and that its psychometric criteria, such as validity and reliability, be verified. The scale was validated on various samples, especially teenagers [10,11] and students [12,13,14,15,16,17,18,19,20]. To our knowledge, the scale has not been validated in the Romanian language. Regarding the analysis of its psychometric properties in the case of a group of teachers, there is only one paper in the international literature, one that analyzes the instrument as to special education teachers [21]. The work of a professor is always a challenge, because he faces several tasks and stress factors. The impact of stress on teachers’ well-being cannot be underestimated. Optimizing the teacher’s well-being is necessary, not only to make him feel good, but also because it influences increased performance in the teaching process and the dynamics of teacher–student relationships [22]. Considering that the well-being of teachers is essential in determining their effectiveness, and that the teaching activity in turn affects the learning and health of students [22,23,24], we aimed to validate the FS in the case of a group of Romanian teachers and to analyze the latter’s level of flourishing. In this framework, we considered elements such as replicating the unifactorial structure of the scale, establishing internal consistency, finding associations with related constructs such as positive affect vs. negative affect and well-being considered from the hedonic perspective, and, finally, the determination of relationships between these items through network analysis.

## 2. Description of the Flourishing Scale (FS)

The FS assesses the optimal functioning level using items that reflect essential components of well-being based on various humanistic psychology theories [2,3,25]. Translated and adapted to various cultural contexts, the FS showed good psychometric properties [4,12,13,14,18,20,26,27,28]. Its eight items refer to purpose and meaning; supportive and rewarding relationships; engagement and interest; contribution to the well-being of others; feelings of competence, self-acceptance, and optimism; and respect from others [4]. Seldom are characteristics like engagement, interest, and optimism included in the other well-being measures that are currently used [4]. The total score can vary between 8 (the lowest one) and 56 (the highest one). A high score indicates developed strengths and psychological resources. Respondents perceive themselves positively in various areas of their lives when there are high scores [4]. In all the validation studies [11,13,15,16,21,26,28,29,30,31,32], the FS exhibited a single factor in its structure, which is consistent with the original study of Diener et al. [4] The scale showed good internal consistency: the authors of the FS reported that Cronbach’s α = 0.87 in the case of certain groups of students [4]. Other studies showed that in student samples a high Cronbach coefficient of over 0.80 was maintained [19,26,27,30], and 0.83 was reached in the case of a sample of full-time employees [26]. Test–retest reliability of the FS, rarely assessed in studies, was good (r = 0.71 [4]; r = 0.87 [33]; r = 0.74 [28]; all retests after one month). The scale demonstrated significant correlations with positive psychology measures aiming at optimism, happiness, life satisfaction [13,28,32], gratitude, resilience, hope [27], reflexivity [19], loneliness, self-esteem, self-control [32], positive and negative affect [4,10,13], stress [13,32], and depression [27,32]. However, as the studies show, combining the scale with other instruments for measuring well-being provides a better understanding of the multidimensional phenomenon of well-being [26].

## 3. Materials and Methods

### 3.1. Participants

The data we will refer to in the present study come from processing the answers of a convenience sample of 323 in-service teachers in the pre-university educational system. The sample is proportionally distributed by sex (50.15% males and 49.84% females). At the time of data collection, participants ranged in age from 21 to 68 years (Mean_age_ = 38.44 years old; *SD* = 9.55). All respondents have university degrees, 38.69% have master’s degrees and 4.02% have doctoral degrees. Among them, 13.62% work at the elementary level, 28.79% at the middle level, and 57.58% at the high school level. Regarding their didactic experience, 9.28% have approximately one year of activity, 30.34% fall within a period of 1–10 years, 32.19% have experience in education ranging between 10 and 20 years, and 28.17% have greater than 20 years of experience in the education field. Only 3.71% of participants work and reside in a rural region, compared to the 96.28% who work in an urban area.

### 3.2. Measures

1. *Flourishing Scale—FS* [4] assesses psychological flourishing. It consists of eight items, in response to which the respondents have to show to what extent they agree, using a continuum from *1*—*strong disagreement* to *7*—strong *agreement*. One sample item reads as follows: “I lead a purposeful and meaningful life.” Although there is a Romanian version of the scale on Ed Diener’s personal website (http://labs.psychology.illinois.edu/~ediener/FS.html, accessed on 10 January 2024), we have not identified any studies that validate the scale in the Romanian. As a result, the linguistic and cross-cultural adaptation procedure was adopted [34]. Thus, the scale was translated from English to Romanian by two independent translators, after which the versions were compared, and a synthesis was made. The newly obtained version was translated from Romanian to English by two other English university professors, one of whom is bicultural. This was followed by the comparison of the translations and the generation of a new synthesis which constituted the final version. A pilot study was conducted on a sample of 13 teachers aged 18–29 who were asked to indicate if there were difficulties in understanding the items. They were not included in the final sample.

2. *Mental Health Continuum-Short Form—MHC-SF* [7] consists of 14 items, assessed on a 6-point Likert scale, which evaluate three components of well-being (emotional, psychological, and social), as experienced by the subject over the last month. Emotional well-being (EWB—3 items) entails the presence of emotions and life satisfaction [35]. Psychological well-being (PWB—5 items) entails aspects related to the individual’s psychological functioning and is expressed in affirmations borrowed from the scale and the model devised by Ryff [36]. Social well-being (SWB—6 items) shows how well individuals function in their social life as members of society (example: social integration and contribution) [37]. In the present study, the internal consistency, shown by α = 0.85 [_95%_CI: 0.83–0.86] and ω = 0.85 [_95%_CI: 0.82–0.88], is high. The 3-factor CFA shows the following good values for goodness-of-fit indices: χ^2^/df = 1.94; CFI = 0.94; TLI = 0.93; RMSEA = 0.055 [_95%_CI: 0.041–0.069]; and SRMR = 0.044.

3. *Scale of Positive and Negative Experience—SPANE* [4] is based on hedonic well-being and consists of 12 items, of which six are connected to positive affects (the subscale Spane-Positive) and the remaining six are associated with negative affects (the subscale Spane Negative). From *1—extremely rarely or never* to *5—very frequently or always*, each item is graded. Each subscale has a score ranging from 6 to 30. The affect balance (SPANE-B) was then computed by deducting the negative score from the positive score. The version validated on Romanian subjects was used in the study [38]. In the current study, for Spane-P, α = 0.87 [_95%_CI: 0.85–0.88] and ω = 0.88 [_95%_CI: 0.85–0.90]; for Spane-N, α = 0.84 [_95%_CI: 0.82–0.85] and ω = 0.84 (_95%_CI: 0.81–0.86). Regarding the factorial structure, CFA shows a good value of the following fit coefficients: χ^2^ = 130.833; df = 53; χ^2^/df = 2.46; CFI = 0.96; TLI = 0.95; RMSEA = 0.064 [_95%_CI: 0.050–0.078]; and SRMR = 0.037.

### 3.3. Procedure

The sample of teachers came from two sources. One part (37.15%) was recruited from two master’s programs related to the teaching career at a large university in the country’s capital city; the other part (62.84%) was recruited from two technological high schools also located in the country’s capital city. Data collection was performed for both online by distributing in email campaigns a link to the site at which the set of tools was posted (in the case of 59% of the participants) and via pencil–paper (for the remaining 41% of teachers) within the didactic master’s programs. The completion of the instruments was anonymous, and it took 9–10 min. The respondents’ informed consent was also obtained. Participation in the survey was voluntary and not rewarded. The eligibility criteria of the respondents were as follows: employment with a full contract for an indefinite period, experience of at least one year in the activity, and having the Romanian language as a native language. The exclusion condition was aimed at eliminating teachers who were not tenured and were only substitutes. This study was conducted in full accordance with relevant ethical principles, including the 1975 World Medical Association Declaration of Helsinki, as revised in 2013. Informed consent was obtained from all participants involved in the study. This study received ethical approval from the Ethics Commission for Scientific Research of the National University of Science and Technology Politehnica Bucharest (Reg. No. 3048/16 October 2023). Participants were informed that, in compliance with EU Rules and Regulation 2016/679 on the protection of natural persons in connection with personal data processing, all responses would be used only for scientific purposes.

### 3.4. Data Analysis

First of all, the sample size required for the study was determined using the calculator developed for structural equation modeling [39]. The sample sizes were calculated using the following parameters: expected effect size of 0.30, desired statistical power level of 0.95, probability level of 0.05, six latent variables, and 34 observable variables. The recommended minimum sample size was 236 respondents, and 323 teachers made up the final sample once the data had been collected.

Averages, standard deviations, skewness, and kurtosis were among the descriptive statistics of the items. To test the construct validity, we used confirmatory factor analysis (CFA) with the Unweighted Least Squares (ULS) estimator method recommended for ordinal data in cases in which there is no multivariate normality assumption [40,41]. To this purpose, we employed various fit indices, including the chi-square test (χ^2^), which had to be statistically insignificant [42]. In addition, the relative/normed chi-square test (χ^2^/df), which has a valid value of 3.00 [43], was also used, but we were conscious of the fact that χ^2^ is sensitive to the sample size [42]. We employed a mixture of indices: CFI (comparative fit index), TLI (Tucker–Lewis index), NFI (Bentler–Bonett normed fit index), and GFI (goodness-of-fit index) with a recommended value of 0.95 [44]. The values of the root mean square error of approximation (RMSEA) and standardized root mean square residual (SRMR) should be less than 0.08 [42]. Using Cronbach’s α and McDonald’s ω, the internal consistency was evaluated. For these indices, a cut-off level over 0.80 is considered excellent [45]. The factor loading was used to calculate the composite reliability (CR) of the scale and the convergent validity through average variance extracted (AVE). According to Awang et al. [46], the minimal threshold for AVE is 0.50 and that for CR is 0.70. Through correlations with the scores of the MHC-SF and SPANE measures, the external convergent validity of the FS was determined. Network analysis was carried out to identify the central items of the scale and to observe the differences between the male and female groups. For this, the centrality indicators of each node were calculated using the following indices: betweenness, closeness, strength, and expected influence. Bootstrapped resampling subsets of participants were used to estimate the stability. A correlation stability coefficient over 0.50 is considered to be a good value [47]. The data were analyzed with the SPSSv23 (IBM, New York, NY, USA) and JASP 0.16.10 (University of Amsterdam, Amsterdam, The Netherlands) programs.

We hypothesized that the FS would have a one-factor structure and that the score would be positively and significantly associated with the SWLS score and the MHC-SF scores and their subscales.

## 4. Results

### 4.1. Descriptive Analysis of the Data

The descriptive statistics were provided as an average with a standard deviation, and skewness and kurtosis indicators were used to calculate the normality of the data (Table 1). By comparing the absolute value to the standard error, it was possible to calculate the Z scores for skewness and kurtosis. It was concluded that the data had a non-normal distribution, because the Z score was not between −3.29 and 3.29, which would correspond to a normal distribution for samples of 50–300 subjects [48]. The average for flourishing is M = 48.66 (*SD* = 5.74; median = 50). The highest average scores are the scores for items related to feelings of competence (M = 6.27—I am competent and capable in the activities that are important to me) and interest in daily activities (M = 6.26—I am engaged and interested in my daily activities), and the lowest one is for the item related to supportive social relations (M = 5.75—My social relationships are supportive and rewarding).

No gender differences were obtained in relation to flourishing (Mean_males_ = 49.20; *SD* = 5.02; Mean_females_ = 48.36; *SD* = 6.31; Mann–Whitney U = 12591.70; *p* = 0.591).

### 4.2. Reliability

We employed the bootstrapping approach (2000 resamplings) because the assumptions of multivariate normality are not valid (Mardia’s coefficient = 75.71; critical ratio—c.r. = 53.78). Table 1 shows the α and ω consistency coefficients, the values of which are excellent, namely 0.89. In addition, in the case of male and female subsamples, we identified high values for the two coefficients: the female group (α = 0.89 [_95%_CI: 0.87–0.91] and ω = 0.89 [_95%_CI: 0.87–0.92]) and the male group (α = 0.87 [_95%_CI: 0.84–0.90] and ω = 0.87 [_95%_CI: 0.84–0.91]). The values of the ω coefficient recalculated for the rest of the items after the elimination of each item were between 0.87 and 0.88.

### 4.3. Construct Validity

Because the FS showed a one-factor structure in all cultural contexts, we replicated directly the one-factor model, which proved to have excellent statistic fit: χ^2^ = 27.889; df = 20; χ^2^/df = 1.39; CFI = 0.99; TLI = 0.99; NFI = 0.98; GFI = 0.99; RMSEA = 0.035 [_90%_CI: 0.000–0.063]; SRMR = 0.058 (Table 2). 

**Figure 1:**
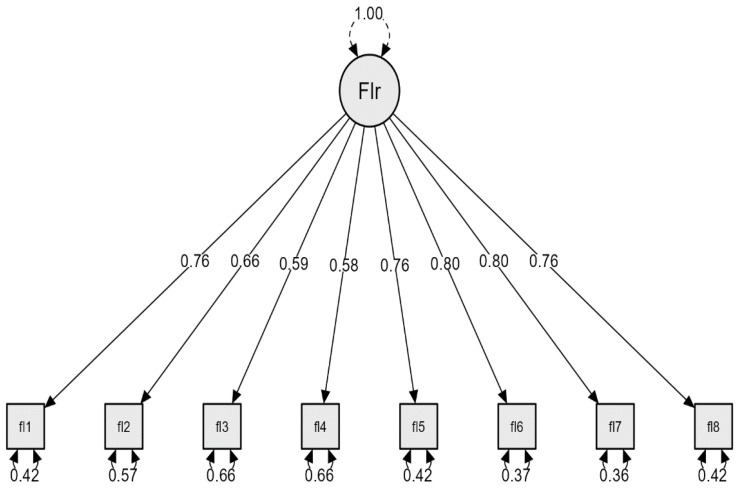
The confirmatory factor analysis for the Flourishing Scale (FS).

The figure highlights the standardized values of the factor loading. The factor loading is between 0.58 and 0.80, a range which is over the minimum requirement of 0.50 considered significant for an item [49], as shown in Figure 1. The corrected item total-correlations are all positive and are between 0.56 and 0.73.

### 4.4. Convergent Validity

We considered the estimated standardized (λ) and standard errors of measurement (ε) obtained through CFA to calculate AVE and CR. Both AVE (0.51) and CR (0.89) are above the minimum cut-off levels of 0.50 and 0.70, respectively [46].

### 4.5. Validity and Intercorrelations

As evidence of convergent validity, the Flourishing Scale (FS) uncovers positive correlations with the instruments of the MHC-SF, together with its dimensions: emotional, psychological, and social well-being (r between 0.41 and 0.66), and SPANE, considering positive affect and balance affect (r = 0.47 and 0.46, respectively). A negative correlation was obtained between the FS and the negative affect subscale (r = −0.32) (all at *p* < 0.01), thus proving the divergent validity of the FS (Table 3). The association with gender does not show significant correlations.

### 4.6. Network Analysis

**Figure 2:**
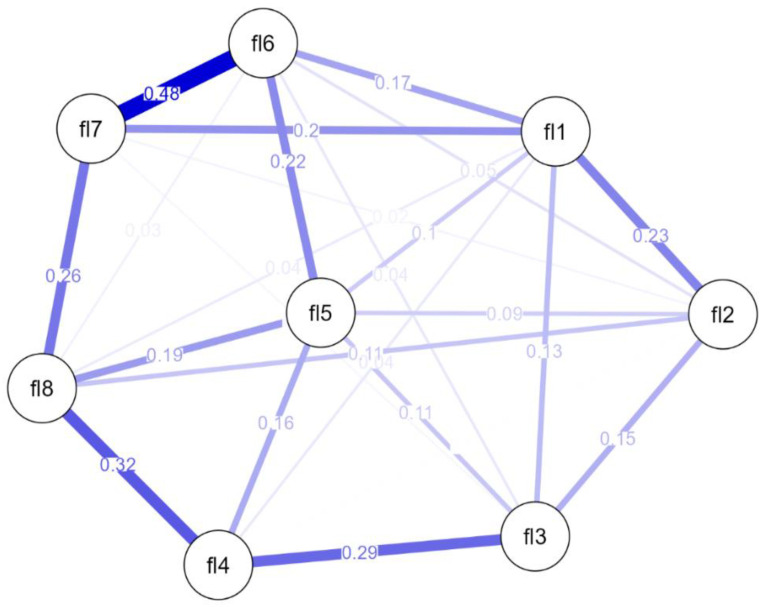
Network analysis of the Flourishing Scale (FS). The numbers on the edges correspond to the edges’ weights; fl1–fl8—items from the FS; Stronger edges are indicated by thicker and shorter lines.

In instrument validation, the perspective of factor analysis can be combined with network analysis, with the intention of gathering further information [50]. Therefore, we performed an estimated network analysis in which variables were represented by nodes and the connections by edges, to detect which items had a central role in the scale. Since the data are not normally distributed, we used the nonparanormal transformation [47]. As a result of the network analysis, we obtained a single cluster in which all nodes are associated with each other (Figure 2). 

**Figure 3:**
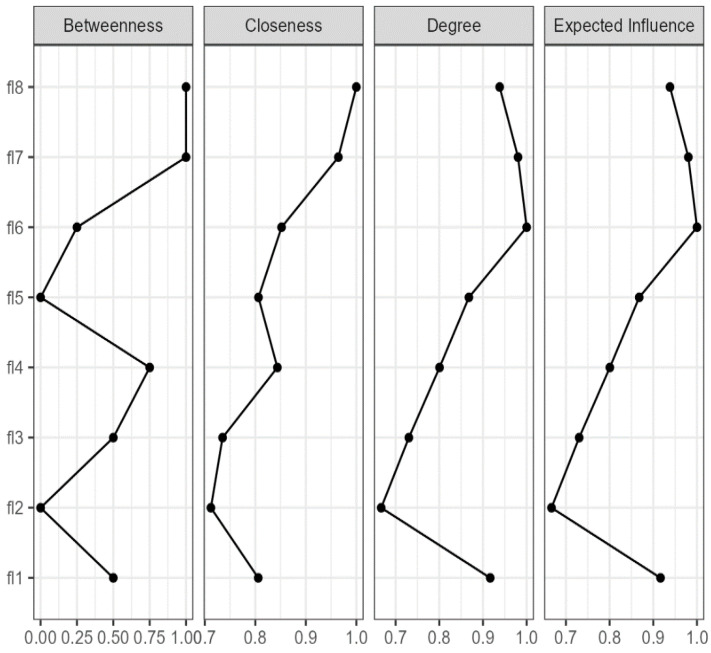
Centrality indices of the network for the Flourishing Scale (FS) items.

Item 6 (self-acceptance), item 7 (optimism), and item 8 (respect) have the highest indicators of centrality (assessing betweenness, closeness, and strength), and item 2 (supportive social relationships), the lowest (assessing betweenness and strength) (CS stability coefficient = 0.60) (Figure 3). A separate analysis was performed for each gender. For the male subsample, the most influential nodes in the network are item 7 (optimism for the future), item 8 (other people’s respect) and item 1 (purpose and meaning of life), and the lowest score for centrality is shown for item 3 (interest in daily activities). In the female subsample, item 8 (other people’s respect), item 6 (self-acceptance and good life), and item 1 (purpose and meaning of life) show high centrality, while item 2 (supportive social relationships) has a low centrality. The evaluation of the connectivity between the nodes shows that the strongest correlation is between items 6 and 7 (0.48), followed by the one between items 8 and 4 (0.32).

## 5. Discussion

The aim of this study was to examine the psychometric properties of the FS instrument in the case of a group of teachers. The results demonstrated that the scale is highly reliable (α and ω = 0.89), a finding similar to those of other studies that have adapted the instrument to other languages, [4,13,29,32,51,52,53], which is suggestive of the high homogeneity of the items. Future studies should examine whether the values of coefficients are stable in other samples of subjects. In addition, AVE = 0.51 and CR = 0.89 are good values, which indicates that they are over the recommended minimum levels. Similar to all prior validation studies, CFA supports the one-factor structure of the scale. The goodness-of-fit indicators demonstrated that the one-factor structure of FS has an appropriate model fit.

The convergent validity of the FS is proved through positive correlations and with values over 0.40 (*p* < 0.001) relative to the scores for two other instruments that operationalize components of well-being (MHC-SF and SPANE). In light of the shared aspects of positive psychology, it was expected that the FS would be related to their respective scales. In this sense, the results are in line with prior research [4,10,13,22].

The network analysis, which, to our knowledge, was here carried out with reference to the FS for the first time, shows a single cluster, which is in line with the factorial structure resulting from CFA. The highest centrality is that of the items which refer to self-acceptance, optimism and respect, whereas the items referring to supportive social relationships have the lowest centrality. For the subsamples differentiated according to gender, the network analysis suggests that, in the case of males, flourishing is related to optimism and respect, while flourishing is weakly associated with involvement and interest in daily activities. In the case of females, flourishing consists of self-acceptance and respect, and social relationships are less included. In general, the results of gender studies are rather equivocal as to flourishing [54]. Our finding is contrary to the one found by Arrosa and Gandelman [55], which considers that, in the case of females, flourishing is due to optimism, but it is consistent with studies that demonstrated the idea that social support and the support of one’s friends are predictors of males’ flourishing, while this is not the case for females’ flourishing [56]. With a very high probability, it can be said that these differences are due to the cultural differences between the samples.

The second purpose of the study was to examine the level of flourishing in the case of the group mentioned. It is essential to be aware of and to evaluate the teachers’ well-being, given that this has immediate effects on their professional development and the quality of the pedagogical activity [57], and also on the quality of work life [58]. A teacher’s academic optimism predicts cognitive and scholastic involvement and, implicitly, the educational aspirations of students [59]. Several studies have analyzed teachers’ well-being [23], but flourishing was rarely examined in these cases. As to the total flourishing score, we first and foremost had in mind the reference point proposed by Diener et al. [4], namely, an average value over 49.00 in the 85th percentile, and, secondly, in comparison with other groups of teachers, since we can cautiously compare individuals from various cultural contexts. In the present case, the analysis of the average, the median, and the percentiles of this group suggest that the analyzed teachers’ flourishing is above average. However, a comparison with other results obtained from the analysis of teacher samples shows that the average flourishing in the present study is not very high. For example, Johnsi Priya [60] found an average of 60.20 (*SD* = 15.34) for secondary teachers, which decreases in the case of rural teachers. Some research shows that the state of flourishing is greater in the case of females [17], while others consider that the state of flourishing is more developed in the case of males [61]. After evaluating the gender differences (Mann–Whitney U), the present research found no differences regarding the total flourishing score in the analyzed group. This aspect is similar to the findings of other studies [60]. Another result of the study highlights that teachers with an optimistic attitude and positive feelings towards themselves will have more chances to experience the flourishing state. This finding correlates with other research conducted on Romanian pre-university teachers which demonstrates that their well-being is closely related to optimism and self-efficacy [62,63]. 

It is necessary to consider the usual limitations of this study when interpreting the findings. The limitations of the study are related to the sample. Although built symmetrically genderwise, most participants come from the urban area compared to the small number of teachers in rural areas. Obviously, results cannot be generalized. Second, the data were based on self-assessment, and they can be influenced by social desirability. Another limitation consists in the absence of extended variables that can help to understand the factors that affect the level of teachers’ flourishing. Since the study used a cross-sectional design, it should be mentioned that another limitation is the absence of longitudinal validity.

## 6. Conclusions

The conclusion we have drawn from the findings of the study is that this version of the Flourishing Scale is a valid, reliable instrument that can be used to examine teachers’ flourishing level, an examination which is very necessary due to the impact that flourishing can have on individual performance. The optimal functioning of teachers should be considered in educational policies as being relevant to the basic values of educational organizations. The use of valid tools for measuring flourishing should be a relevant concern for school psychologists who work with teachers, in order to monitor the states and feelings of the teaching staff in correlation with other considerations such as the school climate and workplace resources. The use of the Flourishing Scale in the evaluation of teachers’ states in correlation with other instruments addressed to both teachers and students (taking into account that the teacher’s well-being controls the student’s learning) would allow the detection of the determining factors of well-being. Also, researchers can focus on the comparative analysis of groups of teachers with a developed state of flourishing vs. teachers with weak or moderate flourishing. The assessment over time of the teachers’ states of flourishing would lead the research to carry out longitudinal analyses which, together with qualitative research, would help to understand how teachers differ in experiencing well-being. School managers who have the role of helping, nurturing, guiding, and assisting teachers so that they can flourish both in the short and long term could use the scale to identify how teachers are functioning behaviorally within the school. They could realize in a certain situation what the factors are that help teachers to have a higher degree of functioning in the environments in which they work.

In this sense, school organizations must continuously monitor the teachers’ functioning levels and treat this aspect not as a singular process, but rather as a permanent investment to ensure productivity and to retain key members of the teaching staff.

## Figures and Tables

**Table 1:** Descriptive statistics of the items.

Items	M	*SD*	Skw. (Std. Error)	Krt. (Std. Error)	ω If Item Dropped
1. I lead a purposeful and meaningful life.	6.02	1.00	−2.05 (0.13)	7.04 (0.27)	0.87
2. My social relationships are supportive and rewarding.	5.75	1.13	−1.10 (0.13)	1.76 (0.27)	0.88
3. I am engaged and interested in my daily activities.	6.26	0.79	−1.19 (0.13)	1.04 (0.27)	0.88
4. I actively contribute to the happiness and well-being of others.	5.96	0.92	−0.74 (0.13)	0.65 (0.27)	0.88
5. I am competent and capable in the activities that are important to me.	6.27	0.91	−1.86 (0.13)	5.44 (0.27)	0.87
6. I am a good person and live a good life.	6.17	0.92	−1.40 (0.13)	2.61 (0.27)	0.87
7. I am optimistic about my future.	6.13	0.94	−1.69 (0.13)	4.81 (0.27)	0.87
8. People respect me.	6.08	0.95	−1.70 (0.13)	5.15 (0.27)	0.87
𝛼 = 0.89 [_95%_CI: 0.87–0.90]					
ω = 0.89 [_95%_CI: 0.87–0.91]					

M—Mean; *SD*—standard deviation; Skw.—Skewness; Krt.—Kurtosis.

**Table 2:** Confirmatory factor analysis.

Model	χ^2^	df	χ^2^/df	CFI	TLI	NFI	GFI	RMSEA [_95%_CI]	SRMR
One factor	27.889	20	1.39	0.99	0.99	0.98	0.99	0.035 [0.000–0.063]	0.058

**Table 3:** The correlations between variables.

Variables	1	2	3	4	5	6	7	8
1. FL (FS)	–							
2. EWB (MHC-SF)	0.54 **	–						
3. PWB (MHC-SF)	0.66 **	0.65 **	–					
4. SWB (MHC-SF)	0.41 **	0.52 **	0.57 **	–				
5. WB total score (MHC-SF)	0.64 **	0.79 **	0.89 **	0.84 **	–			
6. PA (SPANE)	0.47 **	0.58 **	0.50 **	0.40 **	0.56 **	–		
7. NA (SPANE)	−0.32 **	−0.40 **	−0.36 **	−0.29 **	−0.40 **	−0.43 **	–	
8. BA (SPANE)	0.46 **	0.57 **	0.40 **	0.40 **	0.57 **	0.82 **	−0.86 **	
9. Gender	−0.06	0.03	0.01	0.04	0.03	0.02	0.09	−0.04

FL—flourishing; EWB—emotional well-being; PWB—psychological well-being; SWB—social well-being; WB—well-being; PA—positive affect; NA—negative affect; BA—balance affect. The correlations with gender are point-biserial correlations. ** Correlation is significant at the 0.01 level (two-tailed).

## Data Availability

The data presented in this study are available from the corresponding authors upon reasonable request.
